# Current Status and Challenges in Anti-Hepatitis B Virus Agents Based on Inactivation/Inhibition or Elimination of Hepatitis B Virus Covalently Closed Circular DNA

**DOI:** 10.3390/v15122315

**Published:** 2023-11-25

**Authors:** An-Qi Zhuang, Yan Chen, Shan-Mei Chen, Wen-Cheng Liu, Yao Li, Wen-Jie Zhang, Yi-Hang Wu

**Affiliations:** Zhejiang Provincial Key Laboratory of Biometrology and Inspection & Quarantine, Department of Pharmacy, College of Life Sciences, China Jiliang University, Hangzhou 310018, China

**Keywords:** hepatitis B virus (HBV), covalently closed circular DNA (cccDNA), anti-HBV agents, targeting HBV cccDNA, attenuating HBV cccDNA function, epigenetic drugs, gene editing

## Abstract

There has been over half a century since the discovery of hepatitis B virus (HBV) to now, but approximately 300 million patients with chronic hepatitis B (CHB) still live in the world, resulting in about one million deaths every year. Although currently approved antivirals (e.g., nucleoside analogues) are effective at reducing HBV replication, they have almost no impact on the existing HBV covalently closed circular DNA (cccDNA) reservoir. HBV cccDNA is a critical obstacle to the complete elimination of the virus via antiviral therapy. The true cure of HBV infection requires the eradication of viral cccDNA from HBV-infected cells; thus, the development of new agents directly or indirectly targeting HBV cccDNA is urgently needed due to the limitations of current available drugs against HBV infection. In this regard, it is the major focus of current anti-HBV research worldwide via different mechanisms to either inactivate/inhibit (functional cure) or eliminate (complete cure) HBV cccDNA. Therefore, this review discussed and summarized recent advances and challenges in efforts to inactivate/silence or eliminate viral cccDNA using anti-HBV agents from different sources, such as small molecules (including epigenetic drugs) and polypeptides/proteins, and siRNA or gene-editing approaches targeting/attenuating HBV cccDNA via different mechanisms, as well as future directions that may be considered in efforts to truly cure chronic HBV infection. In conclusion, no breakthrough has been made yet in attenuating HBV cccDNA, although a number of candidates have advanced into the phase of clinical trials. Furthermore, the overwhelming majority of the candidates function to indirectly target HBV cccDNA. No outstanding candidate directly targets HBV cccDNA. Relatively speaking, CCC_R08 and nitazoxanide may be some of the most promising agents to clear HBV infection in small molecule compounds. Additionally, CRISPR-Cas9 systems can directly target HBV cccDNA for decay and demonstrate significant anti-HBV activity. Consequently, gene-editing approaches targeting HBV cccDNA may be one of the most promising means to achieve the core goal of anti-HBV therapeutic strategies. In short, more basic studies on HBV infection need to be carried out to overcome these challenges.

## 1. Introduction

Hepatitis B is caused by hepatitis B virus (HBV), a small hepatotropic DNA virus that replicates via reverse transcription, resulting in hepatocellular necrosis and inflammation. As a partially double-stranded DNA virus of the *Hepadnaviridae* family, HBV DNA replication begins with the conversion of relaxed circular DNA (rcDNA) into covalently closed circular DNA (cccDNA) in hepatocellular nuclei [1,2]. Chronic hepatitis B (CHB) is defined as the persistence of hepatitis B surface antigen (HBsAg) for six months or more. CHB remains a major public health problem worldwide, as it can cause liver cirrhosis and hepatocellular carcinoma (HCC) [3]. Currently, approximately two billion people have been infected by HBV, and about 300 million persons are living with CHB infection worldwide. Of those, up to one million people are expected to die annually from complications directly related to their CHB infection [4]. Owing to the fact that viral cccDNA functions as a minichromosome in the nuclei of HBV-infected cells to persist and serve as a template for transcription into viral mRNAs, the true cure for chronic HBV infection necessitates the elimination of viral cccDNA [5]. Although currently available nucleoside analogues (such as lamivudine, telbivudine, tenofovir, and entecavir) can control HBV replication, they are seldom curative and require lifelong treatment for most patients due to viremia often rebounding after therapy cessation. The main reason is that these nucleoside analogues cannot inhibit HBV cccDNA transcription and suppress only a late stage in the HBV life cycle (reverse transcription step) [6]. 

Chronic HBV infection greatly enhances the risk for terminal liver disease. The ultimate goal of therapy for CHB is to reduce the load of HBV cccDNA or permanently silence transcription from HBV cccDNA and ensure sustained activation of an adaptive immune response that prevents the reactivation and spread of residual HBV in the liver. However, none of the available drugs target HBV cccDNA, which is the key obstacle in HBV infection eradication [5]. Apparently, eliminating HBV cccDNA is at the heart of a cure for CHB. However, major treatments for HBV infection, including the uses of interferon-α and nucleotide analogs, are rarely achieved due to an inability to disrupt HBV cccDNA and an inadequate host immune response [7]. Therefore, there remains an urgent need to develop new strategies to completely cure CHB by targeting or attenuating HBV cccDNA in HBV-infected cells. Currently, developing drugs and identifying mechanisms to either eliminate (complete cure) or inactivate (functional cure) HBV cccDNA have become a major focus of HBV research worldwide. While direct-targeting HBV cccDNA strategies are still largely at the preclinical stage of development, capsid assembly modulators and immune-based approaches against HBV cccDNA have reached the clinical phase. This review discussed and summarized recent advances and challenges in efforts to eliminate or inhibit/silence HBV cccDNA using anti-HBV agents from different sources, such as small molecules (including epigenetic drugs), peptides and proteins, and via siRNA or gene-editing approaches (e.g., zinc-finger nuclease, transcription activator-like effector nuclease, and CRISPR/Cas9 systems) targeting or attenuating HBV cccDNA, as well as future directions for the eradication of HBV cccDNA that could be considered in efforts to cure CHB.

## 2. Silence or Elimination of HBV cccDNA

### 2.1. Small Molecules for Epigenetically Silencing HBV cccDNA

As a minichromosome that is inherently stable, viral cccDNA in HBV-infected cells is extremely difficult to clear. Silencing its activity via epigenetic modifications with epigenetic drugs may represent a promising therapeutic strategy to combat CHB. HBV X (HBx) protein has an important role in regulating HBV cccDNA transcription. HBx binds to HBV cccDNA and plays a vital role on the activation of viral gene transcription, leading to high-level HBV replication. In contrast, if HBx binding to HBV cccDNA is inhibited, it will lead to the suppression of viral transcription and replication by modifying the epigenetic regulation of HBV cccDNA function with host restriction factors. HBV core (HBc) inhibitors targeting HBV cccDNA-bound HBc may alter the epigenetics of HBV cccDNA, and in combination with HBx inhibitors, this could lead to a stronger synergistic effect on HBV cccDNA transcriptional silencing and HBV replication [8].

#### 2.1.1. Rapamycin

**Figure 1:**
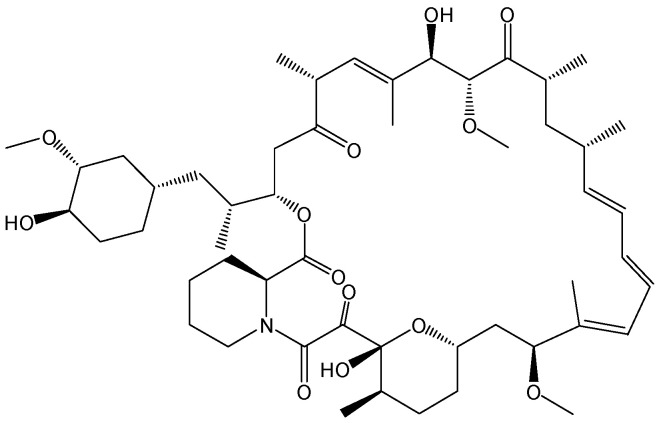
Chemical structure of rapamycin.

Transcriptional silencing of HBV cccDNA is a promising strategy. HBx plays a vitally important role in maintaining the transcriptional activity of HBV cccDNA, and it is a potential target for blocking the transcription of HBV cccDNA. To screen anti-HBV epigenetic drugs by silencing HBV cccDNA, the macrolide compound rapamycin (Figure 1), which has been employed in clinical settings to prevent rejection after organ transplantation, was found to be able to markedly reduce HBx protein expression. Moreover, rapamycin inhibited HBV DNA, HBV RNA, and HBV cccDNA transcription levels in HBV-infected cells and a recombinant HBV cccDNA mouse model. Mechanistic studies have demonstrated that rapamycin can reduce the stability of the HBx protein by promoting its degradation. In addition, HBx deficiency can abrogate the inhibition of HBV cccDNA transcription induced by rapamycin. In short, rapamycin, which targets HBx to block HBV cccDNA transcription, inhibits HBV replication [9]. 

#### 2.1.2. Dicoumarol

**Figure 2:**
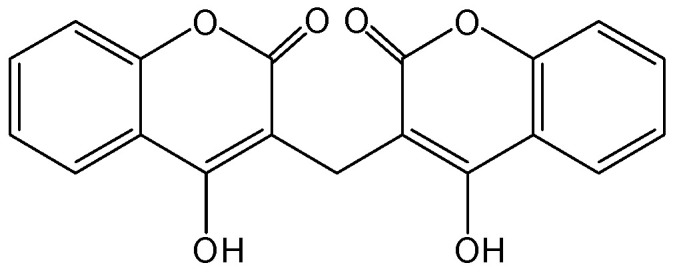
Chemical structure of dicoumarol.

Functional silencing of HBV cccDNA could be achieved by targeting the HBx protein. Dicoumarol was screened from 3840 chemicals using the mutant lentivirus, and it can reduce lentiviral episome DNA and inhibit HBV replication [10]. Dicoumarol (Figure 2), as an inhibitor of quinone oxidoreductase 1 (NQO1), can significantly reduce HBx expression. Moreover, it exhibited potent antiviral activity against HBV DNA, HBV RNAs, HBsAg, and HBc protein in HBV-infected cells and a mouse model with a humanized liver. Mechanistic studies have demonstrated that endogenous NQO1 binds to the HBx protein and protects the HBx protein from 20S proteasome-mediated degradation. Dicoumarol treatment or NQO1 knockdown significantly decreased the recruitment of HBx to HBV cccDNA and suppressed the transcriptional activity of HBV cccDNA, which has been associated with the establishment of a repressive chromatin state. The absence of HBx markedly blocked the antiviral effect induced by dicoumarol treatment or NQO1 knockdown in HBV-infected cells. This suggested that dicoumarol could block HBV cccDNA transcription by promoting HBx degradation [11].

#### 2.1.3. GS-080 and GS-5801

**Figure 3:**
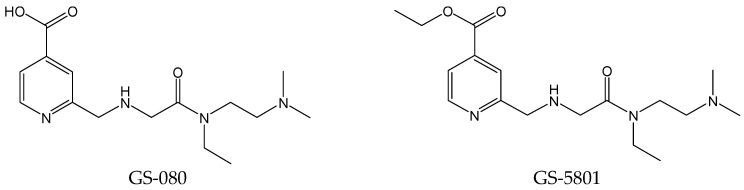
Chemical structures of GS-080 and GS-5801.

To open new avenues for the functional cure of CHB by performing a focused screen of epigenetic modifiers, isonicotinic acids, as a class of the histone lysine demethylase 5 (KDM5) inhibitors with potent anti-HBV activity, were identified. To enhance the liver accumulation and cellular permeability of the most potent KDM5 inhibitor GS-080 (Figure 3), GS-5801 (Figure 3), as an ester prodrug of GS-080, was developed that resulted in improved liver exposure and bioavailability as well as an increased H3K4me3:H3 ratio on chromatin. GS-5801 treatment of HBV-infected primary human hepatocytes decreased the levels of HBV DNA, RNA, and antigen [12]. GS-5801, as a prodrug of a small molecule inhibitor of histone lysine demethylase, aims to silence the transcription of HBV cccDNA. However, it did not exhibit anti-HBV activity in a humanized mouse model [13].

### 2.2. Small Molecules for Suppressing HBV cccDNA

#### 2.2.1. CCC_R08

**Figure 4:**
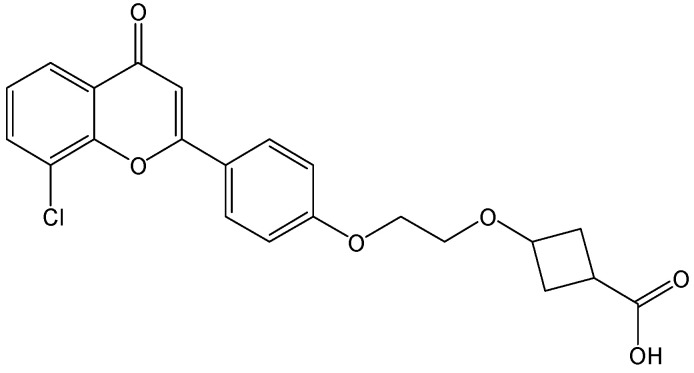
Chemical structure of CCC_R08.

As a small molecule HBV cccDNA inhibitor, CCC_R08 (Figure 4) can specifically reduce HBV cccDNA levels in HBV-infected hepatocytes. More importantly, the level of HBV cccDNA was specifically reduced by CCC_R08, while it did not obviously affect mitochondrial DNA. CCC_R08 treatment led to the sustained reduction of HBsAg and HBV cccDNA levels in the HBV circle mouse model. pgRNA reduction has been found to quantitatively correlate with liver HBV cccDNA reduction. This molecule offers a new approach to completely cure patients with CHB [14].

#### 2.2.2. CCC-0975 and CCC-0346

**Figure 5:**
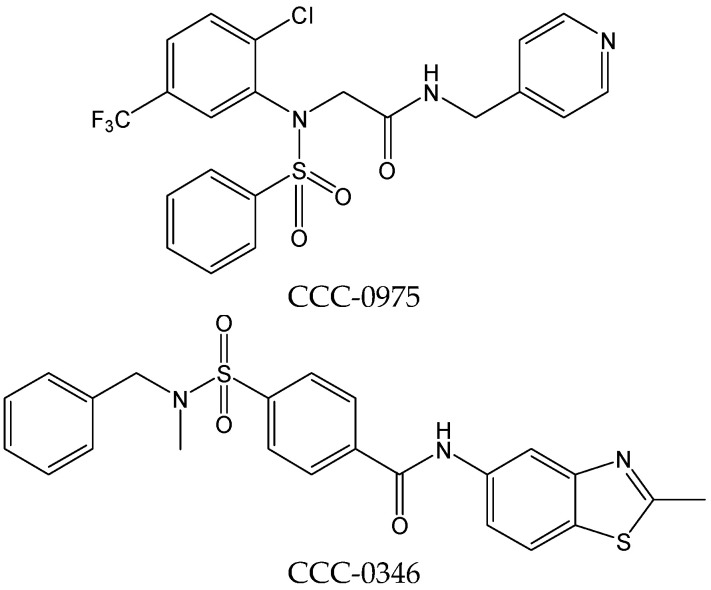
Chemical structures of CCC-0975 and CCC-0346.

CCC-0975 and CCC-0346 (Figure 5), two structurally related disubstituted sulfonamides (DSS), have been confirmed to inhibit the production of HBV cccDNA. Further mechanistic studies have demonstrated that DSS compound treatments neither directly suppressed HBV DNA replication in cell culture nor reduced viral polymerase activity in vitro, but synchronously decreased HBV cccDNA levels and its putative precursor, deproteinized relaxed circular DNA (DP-rcDNA). However, DSS compounds did not enhance the intracellular decay of DP-rcDNA and cccDNA, thus suggesting that the two compounds interfered with rcDNA conversion into cccDNA. DSS compounds potentially served as proof-of-concept drug candidates for development into therapeutics to eliminate HBV cccDNA from CHB [15].

#### 2.2.3. Junceellolide C and Junceellolide B

**Figure 6:**
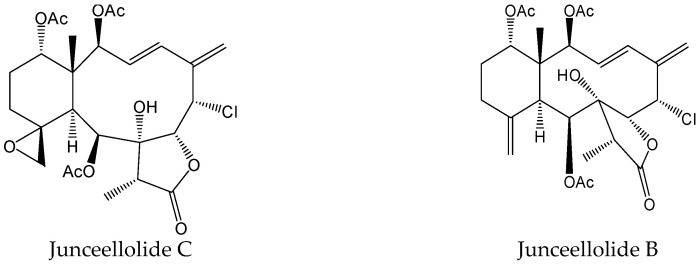
Chemical structures of junceellolide C and junceellolide B.

The gorgonian-derived briarane-type diterpenoids indicated the inhibition of HBV DNA replication in HepAD38 cells. Moreover, a briarane-based scaffold with an 3E,5(16)-diene and a chlorine substitution at C-6 is required for anti-HBV activity. Junceellolide C (Figure 6), as a briarane-type diterpenoid from a gorgonian coral *Ellisella* sp., exhibited efficient reduction against HBV DNA, HBV RNA, and HBeAg production. Meanwhile, it can significantly reduce HBV cccDNA replenishment and promote the existing HBV cccDNA’s degradation [16]. Additionally, junceellolide B (Figure 6) can reduce HBsAg and HBeAg production in HBV-infected HepG2-Na(+)-dependent taurocholate cotransporting polypeptide (NTCP) cells. Moreover, it can still significantly decrease the secreted HBV DNA, HBV RNA, and HBeAg levels in HepAD38 cells. Mechanistically, junceellolide B exhibited a potent inhibition of HBV RNA transcription. RNA-seq analysis indicated that junceellolide B can significantly decrease HBV cccDNA-transcripted products and downregulate the expression levels of RNA polymerase II-related host transcription factors [17]. Therefore, junceellolide C and junceellolide B, as the transcription inhibitors of cccDNA, could present promising leads for the development of new anti-HBV agents.

#### 2.2.4. Compound **59**

**Figure 7:**
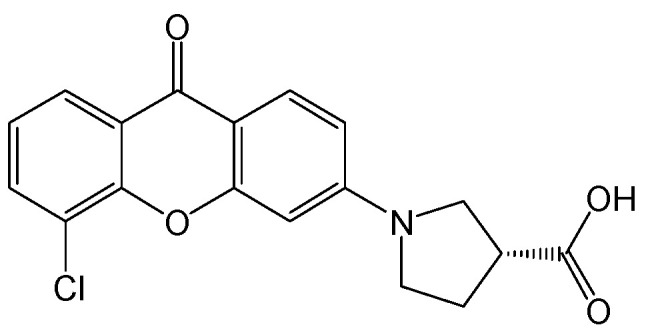
Chemical structure of compound **59**.

Xanthone series hits were discovered to be novel HBV cccDNA reducers through phenotypic screening, and subsequent structure optimization led to the identification of a lead compound with improved pharmacokinetic profiles and anti-HBV activity. A representative compound **59** (Figure 7) was proven to have oral bioavailability and good potency with no cellular toxicity. In an HBV circle mouse model, compound **59** exhibited excellent inhibitory effects on HBV antigens, HBV DNA, and intrahepatic HBV cccDNA production [18].

#### 2.2.5. Epigallocatechin Gallate (EGCG)

**Figure 8:**
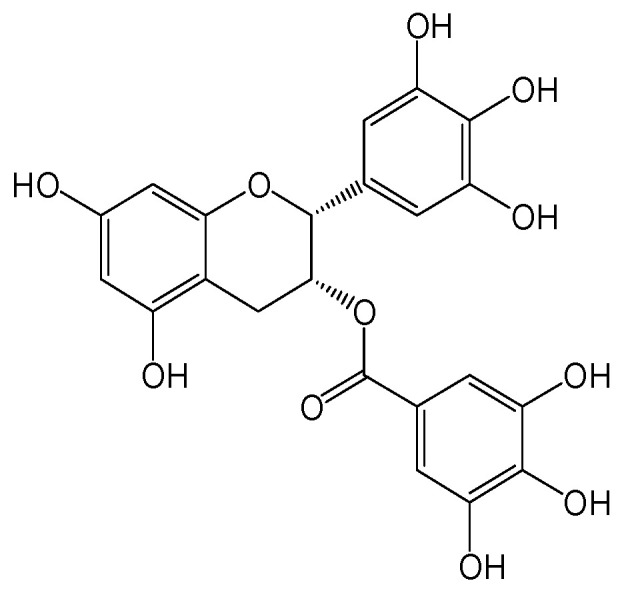
Chemical structure of epigallocatechin-3-gallate.

Epigallocatechin-3-gallate (EGCG) (Figure 8), a major polyphenol from green tea, inhibited HBV replication in HepG2.117 cells by impairing HBV replicative intermediates of DNA synthesis, and such inhibition resulted in a reduction in HBV cccDNA production [19]. Furthermore, EGCG exhibited a potent inhibition of HBV entry and could be used for the prevention of HBV reinfection [20]. In addition, EGCG treatment resisted HBV-induced incomplete autophagy by increasing lysosomal acidification, which is not beneficial for the replication of HBV [21].

#### 2.2.6. Ivermectin

**Figure 9:**
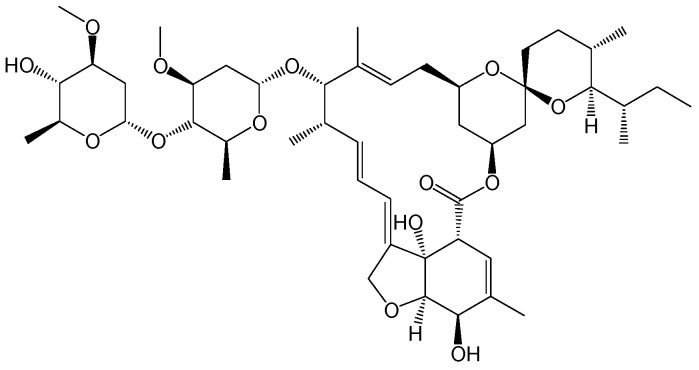
Chemical structure of ivermectin.

Ivermectin (Figure 9), as an antifilarial drug, has been found to suppress HBV production and reduce the levels of several HBV markers (including HBsAg) in HBV-infected human hepatocellular carcinoma cells (HepG2-hNTCP-C4 cells) and humanized mouse hepatocytes (PXB hepatocytes). In addition, it can significantly reduce the expression levels of the HBc protein and the nuclear transporter karyopherin α2 (KPNA2) in the nuclei of HepG2-hNTCP-C4 cells. Furthermore, depleting KPNA1-6 inhibited the production of HBV cccDNA. These findings suggested that ivermectin suppressed the nuclear import of HBV by inhibiting KPNA2 and had the potential to be a novel treatment for chronic HBV infection [22].

#### 2.2.7. 1-[3-(4-Tert-butylcyclohexyl)oxy-2-hydroxypropyl]-2,2,6,6-tetramethylpiperidin-4-ol

**Figure 10:**
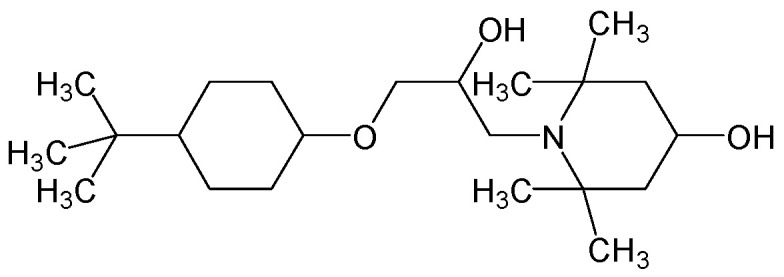
Chemical structure of 1-[3-(4-tert-butylcyclohexyl)oxy-2-hydroxypropyl]-2,2,6,6-tetramethylpiperidin-4-ol.

HBV replication was inhibited by one candidate for an alpha-glucosidase (AG) inhibitor (1-[3-(4-tert-butylcyclohexyl)oxy-2-hydroxypropyl]-2,2,6,6-tetramethylpiperidin-4-ol) (Figure 10). This compound can significantly reduce HBV cccDNA production in fresh human hepatocytes infected with HBV and has a lower anti-AG effect. Its inhibitive effect on HBV replication is via its interaction with the nuclear transcription factor Sp1. Sp1 acts on the multiple regions of HBV to suppress HBV replication. Therefore, identifying candidates that control nuclear transcription factors may facilitate the development of novel therapies against HBV infection [23].

#### 2.2.8. NJK14047

**Figure 11:**
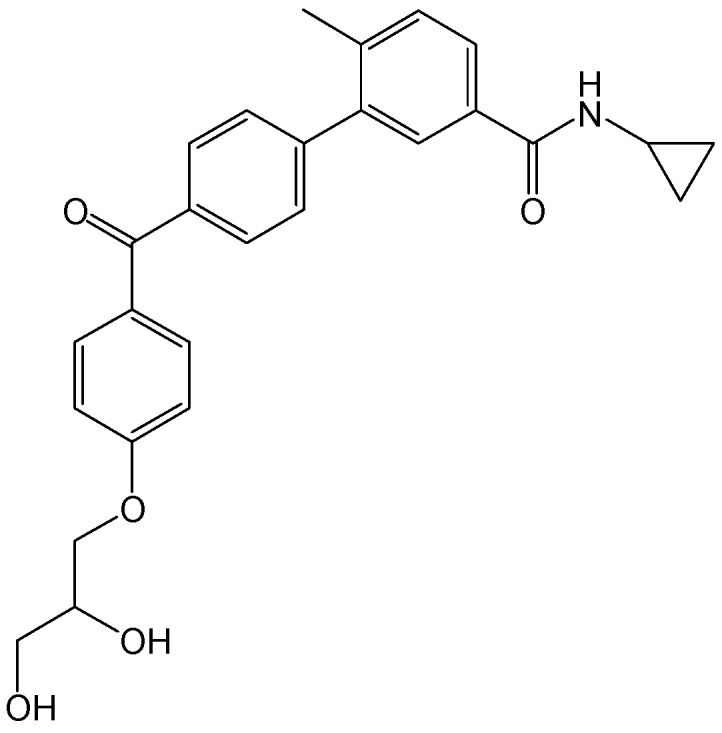
Chemical structure of NJK14047.

Targeting host factors could present an effective antiviral strategy with a low risk of the development of resistance. P38 mitogen-activated protein kinase (MAPK), as a host factor affecting viral replication, has been identified as a potential target against HBV. NJK14047 (Figure 11), as a novel selective inhibitor of p38 MAPK, displayed significant anti-HBV activity, as determined via HBV production as well as HBsAg and HBeAg secretions. NJK14047 showed an efficient suppression of the secretion of HBV antigens and HBV particles from HBV genome-transfected cells and HBV-infected NTCP-expressing human hepatoma cells. Furthermore, NJK14047 treatment was found to result in a marked reduction in HBV cccDNA and pregenomic RNA (pgRNA) in HBV-harboring cells, thus indicating its ability to inhibit HBV replication [24].

#### 2.2.9. 3,4-di-O-CQA and 3,5-di-O-CQA

**Figure 12:**
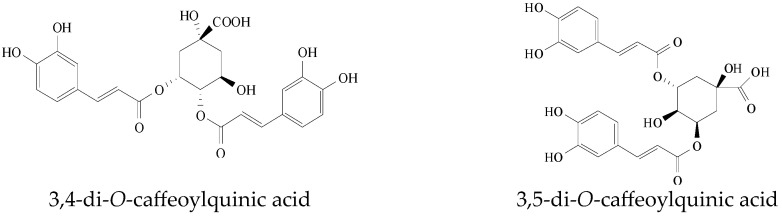
Chemical structures of 3,4-di-*O*-CQA and 3,5-di-*O*-CQA.

The caffeic acid derivatives 3,4-di-*O*-CQAb and 3,5-di-*O*-CQA (Figure 12) from *Laggera alata* significantly inhibited the expression levels of HBsAg and HBeAg, with inhibitive rates of 72.90% and 81.01%, and 86.90% and 89.96%, respectively. Moreover, 3,4-di-*O*-CQA was found to significantly decrease HBV cccDNA content and markedly upregulate heme oxygenase-1 (HO-1) expression in HepG2.2.15 cells and HBV transgenic mice [25]. Additionally, 3,5-di-*O*-CQA exhibited a similar effect as that of 3,4-di-*O*-CQA [26]. Due to the destabilization of HO-1 on the HBc protein, this suggested that HO-1 overexpression could be involved in the antiviral activities of two CQAs by decreasing the stabilization of the HBc protein, which blocks the replenishment of cccDNA in the nuclei of HBV-infected cells [25,26].

#### 2.2.10. ABI-H2158

**Figure 13:**
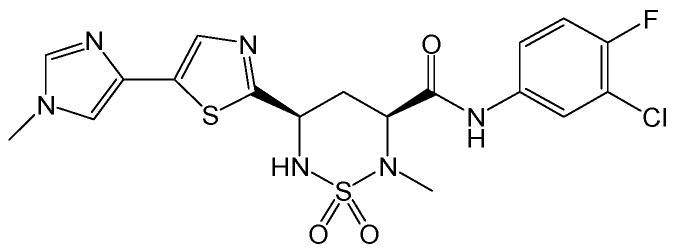
Chemical structure of ABI-H2158.

HBc inhibitors can interrupt multiple steps of the viral replication cycle, including blocking pgRNA encapsidation and prematurely disassembling existing nucleocapsids, thereby preventing them from transporting HBV rcDNA to the hepatocyte nucleus for conversion into HBV cccDNA. ABI-H2158 (Figure 13) is an HBc inhibitor that advanced into phase II clinical trials for the treatment of CHB, but it was not continued due to its hepatotoxicity. ABI-H2158 can inhibit HBV replication by blocking pgRNA encapsidation in induced HepAD38 cells and exhibited a similar potency in primary human hepatocytes and HBV-infected HepG2-NTCP cells. Moreover, it is a pan-genotypic HBV inhibitor across HBV genotypes A-E. This compound can still potently block the formation of HBV cccDNA in de novo HBV infections in HepG2-NTCP and primary human hepatocyte assays. These findings suggest that ABI-H2158 possesses dual mechanisms of action for inhibiting both early and late steps of the HBV replication cycle [27].

#### 2.2.11. Pimobendan (Pim)

**Figure 14:**
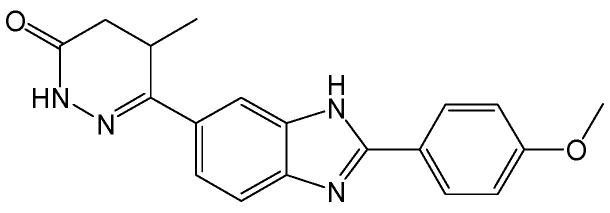
Chemical structure of pimobendan (Pim).

Pimobendan (Pim) (Figure 14), as detailed from the FDA-approved drug library, was identified to exhibit a powerful antiviral activity. Its inhibitory effects on HBsAg as well as other HBV markers were validated in HBV-infected cells and HBV transgenic mice. Mechanistically, Pim is an inhibitor of HBV transcription by suppressing HBV promoters to reduce HBV RNAs and HBsAg levels. In short, Pim is a transcription inhibitor of HBV cccDNA, thereby inhibiting HBsAg and other HBV replicative intermediates both in vitro and in vivo. This compound may provide a potentially promising lead for the development of new agents against HBV infection [28].

#### 2.2.12. ABI-H0731

**Figure 15:**
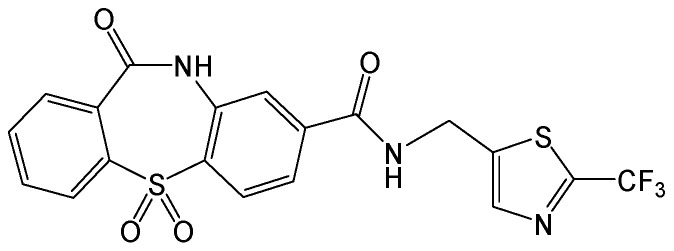
Chemical structure of ABI-H0731.

ABI-H0731 (Figure 15), as the first generation of HBc protein inhibitors, has been demonstrated to exhibit potent antiviral activity in CHB patients in a phase Ib clinical trial, and is currently being further evaluated in phase II clinical studies. ABI-H0731 exhibited the inhibition of HBV DNA replication and HBV cccDNA formation in two de novo infection models. Mechanistically, ABI-H0731, as a direct-acting antiviral agent, targets the HBc protein to prevent HBV pgRNA encapsidation and subsequent DNA replication. In addition, ABI-H0731 disrupted incoming nucleocapsids, causing the premature release of rcDNA before delivery to the hepatocyte nucleus, and thus preventing the formation of new HBV cccDNA [29].

#### 2.2.13. UCN-01

**Figure 16:**
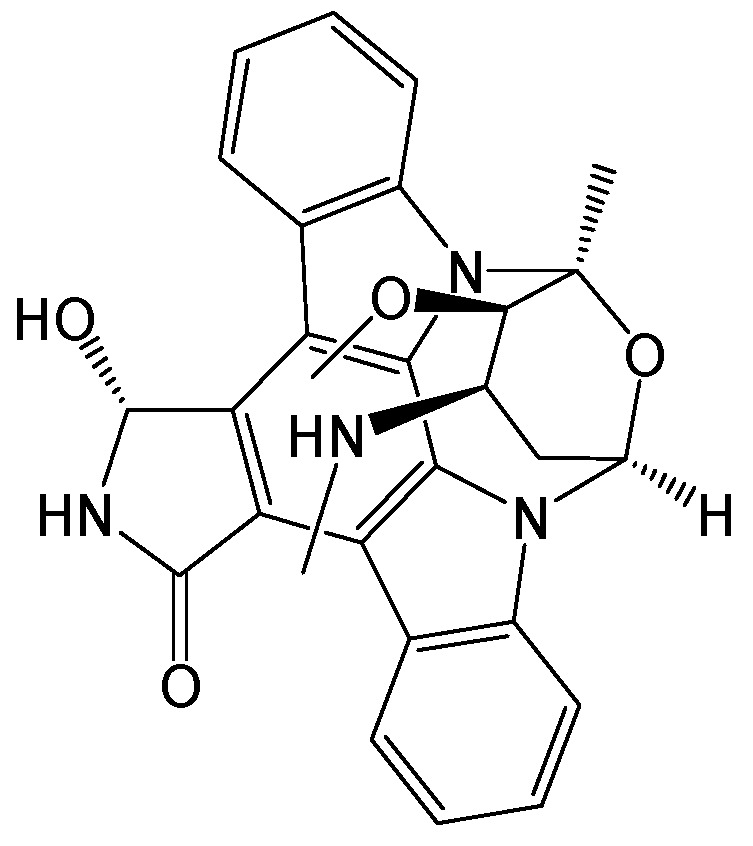
Chemical structure of UCN-01.

Recent studies have suggested that HBV replication can be controlled by the host cell cycle machinery. Therefore, it has been speculated that HBV cccDNA synthesis may be modulated via cell cycle progression. Further research has showed that treatment with UCN-01 (Figure 16), an inhibitor with a broad spectrum activity for cyclin-dependent kinase (CDK) and phosphorylated protein kinase C (PKC) proteins, can greatly reduce HBV cccDNA levels in virus-producing HepAD38 cells. In accordance with HBV cccDNA blockage, the intracellular viral pgRNA and DNA levels, HBc and HBV surface (HBs) protein expression levels, as well as the level of HBeAg secreted in the cell medium were also reduced following UCN-01 treatment [30].

#### 2.2.14. AZD-5438

**Figure 17:**
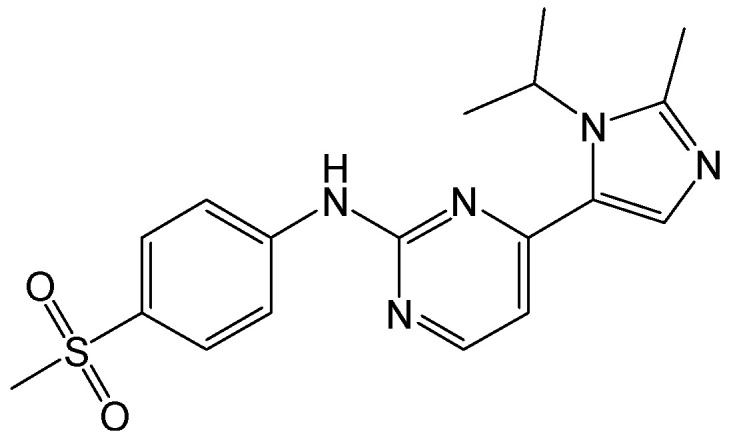
Chemical structure of AZD-5438.

AZD-5438 (Figure 17), as a potent inhibitor of CDK1, CDK2, and CDK9, exhibited antiproliferative activity in human tumor cell lines [31]. Meanwhile, AZD-5438 dramatically reduced the level of HBV cccDNA production in HepAD38 cells in a dose-dependent manner. This indicates that CDK activity is required for HBV cccDNA synthesis. AZD-5438 was found to be able to block intracellular HBV cccDNA synthesis; thus, host CDK activity would be likely required for HBV cccDNA synthesis and could be potentially applied for antiviral drug screening for the development of potent HBV cccDNA inhibitors [30].

#### 2.2.15. Tazarotene

**Figure 18:**
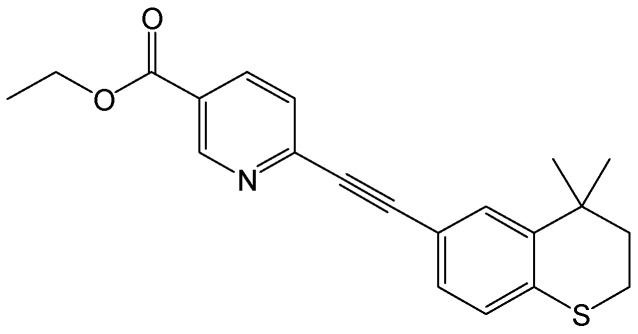
Chemical structure of tazarotene.

Tazarotene (Figure 18), a retinoic acid receptor (RAR) agonist, can reduce HBsAg levels in primary human hepatocytes infected by HBV. This inhibitory effect has also been observed in HBV-infected differentiated HepaRG models, and HBV genotypes A to D were similarly inhibited. HBV cccDNA transcription was repressed by tazarotene, as determined via the HBV cccDNA and RNA levels and the HBV promoter activation. Furthermore, tazarotene can alter a number of gene expressions associated with RAR and metabolic pathways. Anti-HBV activity of tazarotene was found to be significantly attenuated when RARβ was inhibited by a specific antagonist, thus suggesting that tazarotene inhibited HBV, in part, through RARβ. Therefore, RAR agonists, as represented by tazarotene, could be potential anti-HBV candidates [32].

#### 2.2.16. Peretinoin

**Figure 19:**
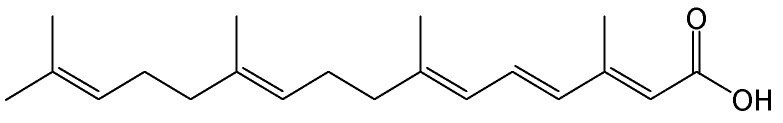
Chemical structure of peretinoin.

Peretinoin (Figure 19) can significantly reduce the levels of intracellular HBV DNA, nuclear HBV cccDNA, and HBV transcript in HepG2.2.15 cells. Mechanistically, peretinoin enhanced the binding of histone deacetylase 1 (HDAC1) to HBV cccDNA in hepatocyte nuclei and negatively regulated HBV transcription, although it increased the expression of HBV-related transcription factors. Moreover, peretinoin can significantly inhibit the expression of sphingosine kinase 1 (SPHK1), an inhibitor of HDAC activity. Meanwhile, the inhibition of HBV replication induced by peretinoin can be cancelled via SPHK1 overexpression in cells. This suggests that peretinoin activates HDAC1 and thereby suppresses HBV replication by inhibiting the sphingosine metabolic pathway [33]. 

#### 2.2.17. Curcumin

**Figure 20:**
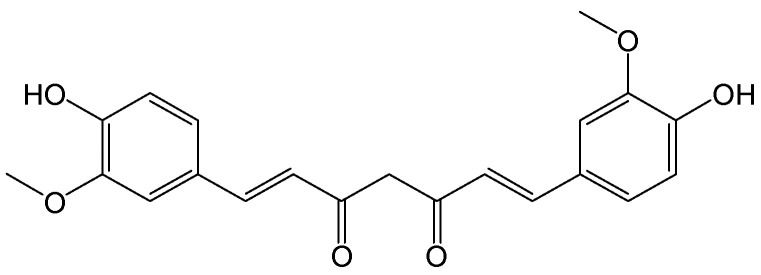
Chemical structure of curcumin.

Curcumin (Figure 20) treatment led to significant reductions in HBsAg and HBeAg expression levels and in intracellular HBV DNA replication intermediates and HBV cccDNA levels in HepG2.2.15 cells. After treatment with 20 μmol/L curcumin for 2 days, compared with levels in non-treated cells, HBV cccDNA and HBsAg levels in HepG2.2.15 cells were decreased by up to 75.5% and 57.7%, respectively. Meanwhile, following treatment with curcumin, histone H3 acetylation levels were decreased in a time- and dose-dependent manner and accompanied by reductions in H3- and H4-bound HBV cccDNA. This suggests that curcumin inhibits HBV replication via the downregulation of HBV cccDNA-bound histone acetylation and has the potential to be a cccDNA-targeting anti-HBV agent [34].

#### 2.2.18. Punicalagin, Punicalin, and Geraniin

**Figure 21:**
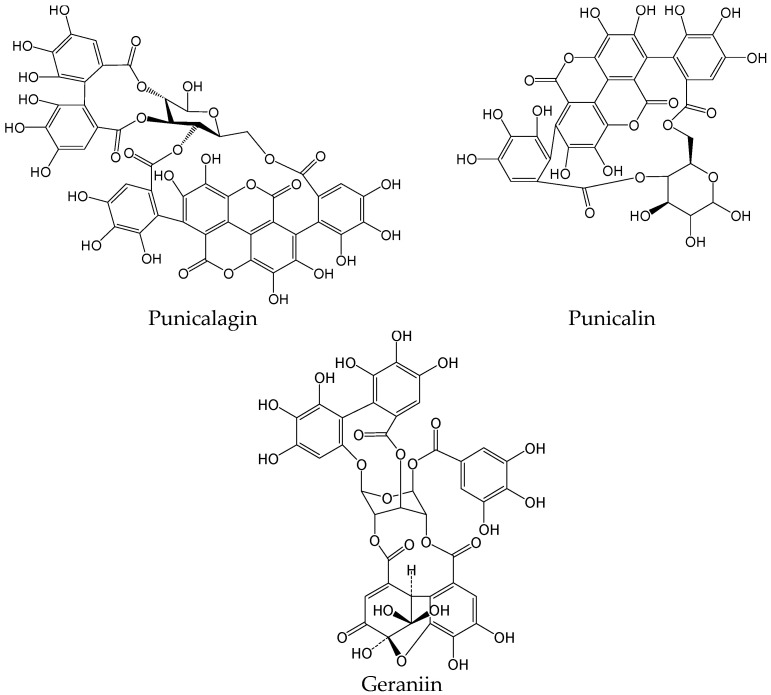
Chemical structures of hydrolyzable tannins (punicalagin, punicalin, and geraniin).

Three hydrolyzable tannins (punicalagin, punicalin, and geraniin) (Figure 21) derived from Chinese herbal remedies significantly reduced the production of HBV cccDNA and HBeAg via a cell-based assay. Moreover, punicalagin did not affect pgRNA transcription, precore/core promoter activity, HBc protein expression, and HBsAg secretion. These tannins can significantly inhibit the establishment of HBV cccDNA and modestly facilitate the degradation of pre-existing HBV cccDNA in the cell-based HBV cccDNA accumulation and stability assay. Collectively, this revealed that three tannins suppress HBV cccDNA production via a dual mechanism by preventing HBV cccDNA formation and promoting HBV cccDNA decay. Therefore, these tannins have the potential to become lead compounds for the development of new agents against HBV infection [35].

#### 2.2.19. Osalmid and YZ51

**Figure 22:**
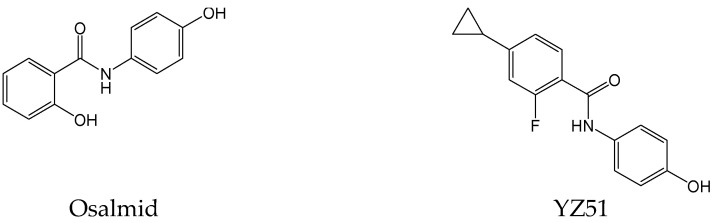
Chemical structures of osalmid and YZ51.

Ribonucleotide reductase (RR) regulates the biosynthesis of deoxyribonucleoside triphosphates in host liver cells, and it has been demonstrated to be essential for HBV replication and HBV cccDNA synthesis. Osalmid (Figure 22), as a potential RR small subunit M2 (RRM2)-targeting compound, significantly inhibited the synthesis of HBV DNA and cccDNA in HepG2.2.15 cells. Furthermore, its derivative 4-cyclopropyl-2-fluoro-N-(4-hydroxyphenyl) benzamide (YZ51) (Figure 22) exhibited higher efficacy than osalmid with more potent RR inhibitory activity. In short, RRM2 may be a potential target against HBV, and osalmid and its derivative YZ51 could be a novel class of anti-HBV candidates [36].

#### 2.2.20. Irbesartan

**Figure 23:**
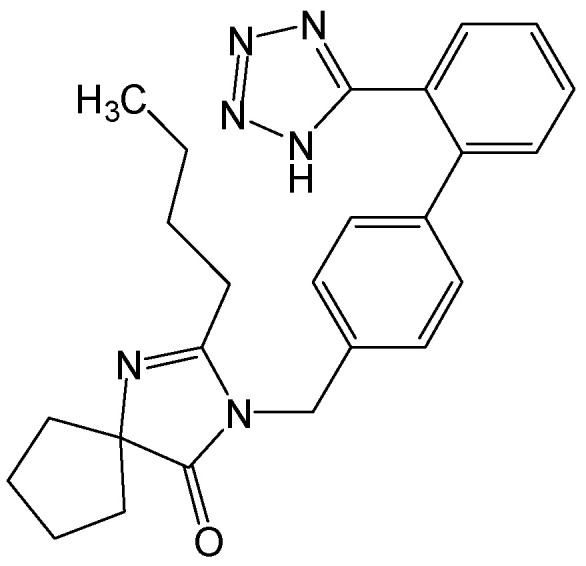
Chemical structure of irbesartan.

Irbesartan (Figure 23) is a clinically approved drug against diabetic nephropathy and hypertension and also a new NTCP-interfering molecule. It effectively inhibited HBV infection with an IC_50_ of 3.3 μM for HBeAg expression in NTCP-overexpressing HepG2 cells named HepG2.N9 susceptible to HBV infection. Irbesartan also efficiently inhibited HBV cccDNA formation and weakly suppressed HBV uptake. This suggests that irbesartan inhibits HBV infection at a post-uptake level prior to the HBV cccDNA formation step, for instance the cell membrane fusion. Based on these findings, irbesartan could present a potential candidate against HBV infection [37].

#### 2.2.21. MLN4924

**Figure 24:**
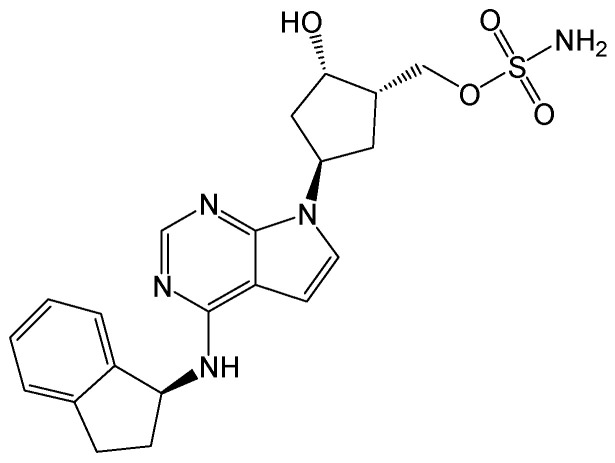
Chemical structure of MLN4924.

MLN4924 (Figure 24) is a selective inhibitor of NEDD8-activating enzyme. Treatment with MLN4924 can effectively suppress HBV DNA, RNA, HBsAg, and cccDNA production in the HBV-expressing Huh7, HepG2.2.15, and HepG2 cells transfected with HBV plasmids and in the mouse models with hydrodynamic injections of pAAV-HBV1.2 plasmids. Mechanistically, by blocking cullinneddyltion and activating ERK, MLN4924 suppressed the expression of several transcription factors required for HBV replication, such as C/EBPα, HNF1α, and HNF4α, leading to a potent blockage in HBV cccDNA and HBV antigen production [38]. MLN4924 profoundly inhibited the transcription of HBV cccDNA, suppressed the production of HBsAg encoded by integrants, and reduced intracellular HBsAg levels (independent of HBx). By employing the HBV-inducible cell line HepAD38 as a model, the dual action of MLN4924 was verified on both HBV cccDNA and integrants with sustained inhibition of HBV markers. Neddylation is required both for the genomic integration of viral DNA and for the transcription of HBV cccDNA [39]. Therefore, blocking neddylation may provide a novel approach for curing CHB. In short, the neddylation pathway could play a critical role in viral DNA integration. Inhibiting this pathway may hold therapeutic promise for patients with CHB [40].

#### 2.2.22. Rnase H Inhibitors 110, 1133, and 1073

**Figure 25:**
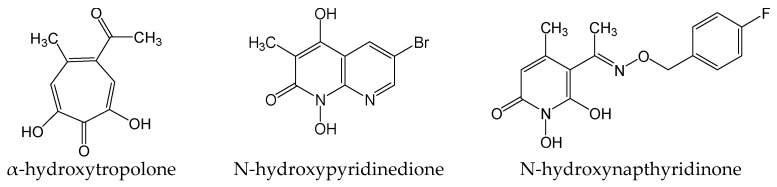
Chemical structures of α-hydroxytropolone, N-hydroxypyridinedione, and N-hydroxynapthyridinone.

HBV ribonuclease H (Rnase H) is an attractive target against viral infection. Three Rnase H inhibitors (α-hydroxytropolone, N-hydroxypyridinedione, and N-hydroxynapthyridinone) (Figure 25) from different chemotypes suppressed the formation of HBV cccDNA by >98% at 5 μM in HBV-infected HepG2-NTCP cells. Furthermore, HBV RNA, extracellular and intracellular DNA, and HBsAg secretion were all robustly inhibited. The great efficacy of these Rnase H inhibitors could be due to blocking HBV cccDNA amplification, which suppresses events downstream of HBV cccDNA formation. These compounds reduced wild-type and lamivudine/adefovir-resistant HBV replication with similar EC_50_, thus indicating that these Rnase H inhibitors do not target HBV reverse transcriptase [41].

#### 2.2.23. Clevudine and ATI-2173

**Figure 26:**
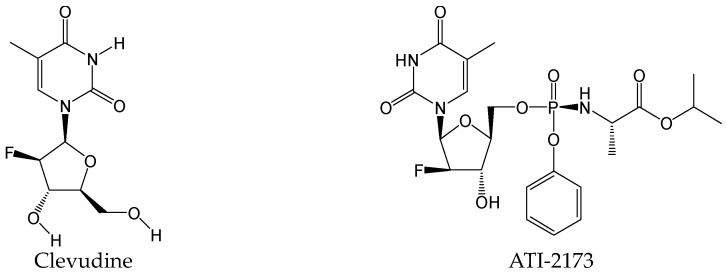
Chemical structures of clevudine and ATI-2173.

Active site polymerase inhibitor nucleotides (ASPINs) can completely inhibit all polymerase functions by non-competitively distorting the HBV polymerase active site. Clevudine (Figure 26), as a first-generation ASPIN, exhibited potent and prolonged HBV suppression in phase II and III clinical trials, but it was found to be associated with reversible myopathy in a small number of patients during long-term treatment. ATI-2173 (Figure 26), as a novel next-generation ASPIN, is similar to clevudine in structure but targets the liver and also exerts robust anti-HBV effects on and off treatment and possesses an improved safety and pharmacokinetic profile by markedly decreasing systemic clevudine exposure. ASPINs have the potential ability to reduce HBV cccDNA. In a phase Ib study in subjects with CHB, serum HBcAg and HBV RNA, both serologic biomarkers for HBV cccDNA, were reduced from baseline after ATI-2173 treatment for 28 days [42].

#### 2.2.24. HAP_R01

**Figure 27:**
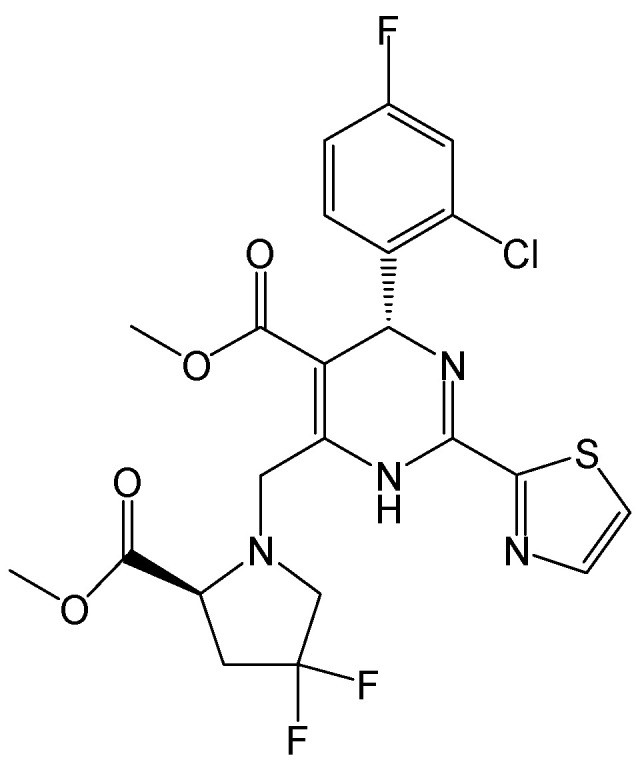
Chemical structure of HAP_R01.

HBc protein allosteric modulators (CpAMs) can prevent correct capsid assembly but may also affect the early stages of HBV infection. HAP_R01 (Figure 27) is a structurally distinct heteroaryldihydropyrimidine (HAP)-type CpAM [43]. It exhibited a significant suppression of HBV cccDNA formation. HAP_R01 can physically alter the mature capsids of incoming HBV particles and affect viral particle integrity. After the purified HBV virions were treated with HAP_R01, their infectivity decreased, thus highlighting the unique anti-HBV activity of CpAMs by targeting the capsids within mature viral particles. Taken together, HAP_R01 perturbed the capsid integrity of incoming HBV particles and decreased their infectivity, thus inhibiting HBV cccDNA formation and preventing HBV capsid assembly [44]. Generally, as the promising inhibitors of HBV replication, HAPs are known to promote the mis-assembly of the HBc protein [45]. Currently, there are two classes of CpAM: the HAPs and the phenylpropenamides (PPAs). HAP_R01 not only reduced HBV DNA levels but also directly inhibited HBeAg secretion via induction of its mis-assembly in vitro and in vivo. Hence, HAP_R01 has the potential to achieve higher rates of anti-HBeAg seroconversion than the approved therapies for chronic HBV infection [46]. 

#### 2.2.25. FIT-039

**Figure 28:**
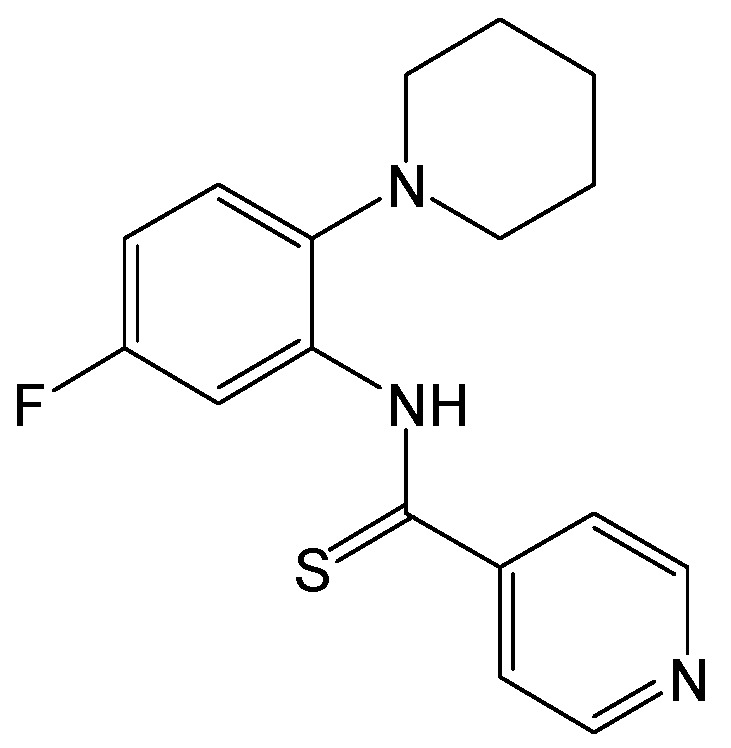
Chemical structure of FIT-039.

FIT-039 (Figure 28) is a CDK9 inhibitor. It is known to inhibit the replication of several DNA viruses, including human immunodeficiency virus (HIV), human herpes simplex virus (HSV), human papilloma virus (HPV), and human adenovirus [47,48,49]. The anti-HBV activity of FIT-039 during the early phase of a viral infection is very prominent, although it does not affect preS1 binding to HepG2/NTCP cells. FIT-039 decreased HBV cccDNA levels in HBV-replicating or HBV-infected cells. These findings indicate that FIT-039 is a promising candidate for the treatment of patients with CHB [50].

#### 2.2.26. Olaparib

**Figure 29:**
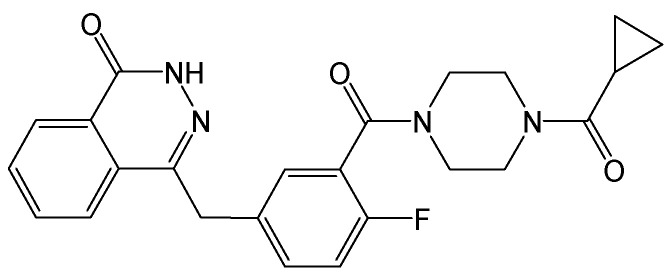
Chemical structure of olaparib.

Olaparib (Figure 29), a clinically available poly(adenosine diphosphate ribose) polymerase (PARP) inhibitor, increased the reduction in HBV cccDNA and pgRNA levels induced via HBV-CRISPR in HBV-infected HepG2-hNTCP-C4-iCas9 cells and primary human hepatocytes. The inhibition of the nonhomologous end joining (NHEJ)-mediated DNA repair machinery promoted the effect of CRISPR targeting HBV cccDNA. The combination of olaparib and CRISPR may present a therapeutic strategy for HBV infection [51].

#### 2.2.27. Vonafexor (EYP001)

**Figure 30:**
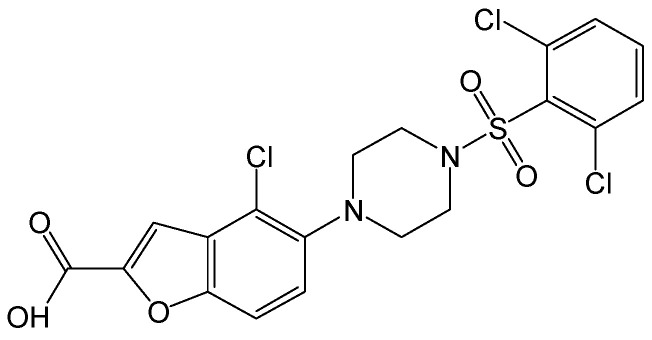
Chemical structure of vonafexor (EYP001).

The nuclear farnesoid X receptor (FXR) can regulate bile acid homeostasis. It is known to be a drug target for metabolic liver diseases and plays an important role in HBV DNA transcription. Treatment with FXR agonists led to the suppression of HBV replication and a decline in HBV proteins, pgRNA levels, and HBV DNA levels. Vonafexor (EYP001) (Figure 30), as a FXR agonist, is a potent inhibitor of HBV cccDNA transcription under clinical development [52,53]. The safety and antiviral effects of EYP001 were evaluated in a total of 73 CHB patients that were enrolled in a two-part, double-blinded, placebo-controlled phase Ib trial. This study found that EYP001 was well tolerated overall and safe, with a decline in HBV markers observed in patients with CHB [54]. 

#### 2.2.28. Nitazoxanide and Tizoxanide

**Figure 31:**
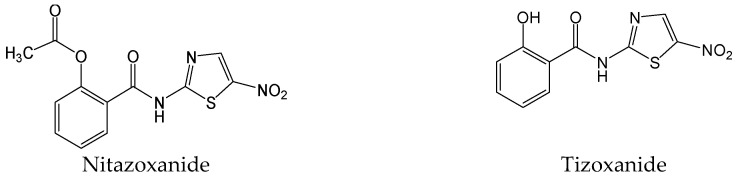
Chemical structures of nitazoxanide and tizoxanide.

Nitazoxanide (Figure 31) is a first-in-class antiprotozoal agent that was licensed in the United States in 2002. It was later reported that nitazoxanide and its active circulating metabolite tizoxanide (Figure 31) exhibited potent inhibitions of HBV replication (HBV DNA, hepatitis B core antigen, HBeAg, and HBsAg) in HepG2.2.15 cells [55]. Nitazoxanide was equally effective at inhibiting the replication of lamivudine- and adefovir dipovoxil-resistant HBV mutants and displayed synergistic interactions with lamivudine and adefovir dipovoxil against HBV [55]. Furthermore, nitazoxanide was still found to encompass a broad spectrum antiviral activity against different viral infections, such as hepatitis C virus, influenza, coronaviruses, rotavirus, norovirus, human immunodeficiency virus, and other viruses, in cell culture assays [56]. Additionally, clinical trials showed a potential role for thiazolides in treating influenza, CHB, chronic hepatitis C, rotavirus, and norovirus gastroenteritis [56].

Mechanistically, nitazoxanide silenced the transcription of HBV cccDNA and decreased viral cccDNA levels slightly by targeting HBx damage-specific DNA-binding protein 1 (DDB1) interaction and significantly restoring Smc5 protein levels in the HBV minicircle system and in the HBV-infected human primary hepatocytes [57]. A pilot clinical trial suggested that nitazoxanide may not only rapidly decrease serum HBV DNA following treatment but also lead to serum HBsAg loss in a significant number of cases, and they are also consistent with previous anti-HBV evaluations in vitro [58]. Nitazoxanide provides novel antiviral mechanisms in treating CHB, and it would be interesting to evaluate the potential of combining nitazoxanide with nucleos(t)ide analogues. Generally, nitazoxanide, as the scaffold for a new class of drugs called thiazolides by targeting an HBV-related viral–host protein interaction, may be a promising new antiviral agent.

### 2.3. Polypeptides/Proteins for Inhibiting HBV cccDNA 

#### 2.3.1. Bulevirtide (MyrcludexB/Hepcludex)

**Figure 32:**
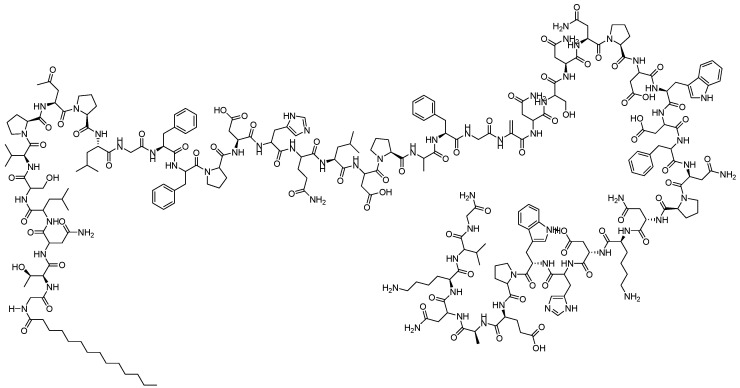
Chemical structure of bulevirtide (myrcludex B).

The lipopeptide myrcludex B (Figure 32) was found to be the first entry inhibitor that can inactivate HBV and hepatitis D virus (HDV) receptors. It competes with HBV for the NTCP, which was identified as the bona fide receptor for HBV and HDV, blocked HBV infection in hepatocytes, and participated in HBV transcriptional suppression [59]. Myrcludex B exhibited an effective inhibition of HBV cccDNA amplification and intrahepatic infection spread [60]. In short, myrcludex B is a first-in-class compound that can block the entry of HBV and HDV into hepatocytes [61]. It plays an important role in the suppression of HBV replication and is a potential drug in phase III clinical trial [62]. In addition, Gilead submitted their biologics license application to the U.S. FDA for bulevirtide (*Hepcludex*), an investigational treatment for the patients with chronic hepatitis delta.

#### 2.3.2. Pam3SCK4 

**Figure 33:**
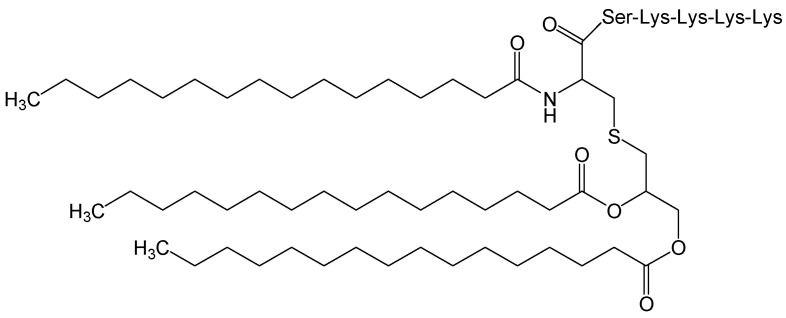
Chemical structure of Pam3CSK4.

The toll-like receptor (TLR)-2 agonist Pam3CSK4 (Figure 33) is a novel immune stimulator. The long-lasting anti-HBV activity of this agonist exhibited a strong reduction in HBV RNA production (inhibition of synthesis and acceleration of decay) and HBV cccDNA levels in HBV-infected hepatocytes and demonstrated the specificity of its action via the TLR1/2-NF-κB canonical-pathway. Flap endonuclease 1 (FEN-1) could be involved in the regulation and inhibitory phenotype of Pam3CSK4 on HBV cccDNA levels. Moreover, the combination of Pam3CSK4 with interferon-α (IFN-α), as a valuable strategy to reduce HBV cccDNA levels, achieved a long-lasting anti-HBV effect. In general, this TLR2 agonist may represent a valuable asset to improve the treatment of CHB patients [63].

#### 2.3.3. IFN-α, IFN-β, and IFN-γ

IFN-α treatment may clear HBV on paper, but it is limited by its systemic side effects. The activation of IFN-α and lymphotoxin-β receptors can upregulate APOBEC3A and APOBEC3B cytidine deaminases, respectively. The HBc protein-mediated interaction with nuclear HBV cccDNA results in cytidine deamination, apurinic/apyrimidinic site formation, and finally HBV cccDNA degradation that prevents HBV reactivation [64]. Since HBV cccDNA can be non-cytolytically degraded via the agents that can upregulate APOBEC3A and 3B, they may represent a critical first step towards the development of a cure for the patients with CHB [65]. IFN-α epigenetically regulates the HBV cccDNA minichromosome by modulating the GCN5-mediated succinylation of histone H3K79 to clear HBV cccDNA. These findings provide a new insight into the mechanism by which IFN-α modulates the epigenetic regulation of the HBV cccDNA minichromosome [66]. Currently, of the drugs available, only IFN-α can potentially target HBV cccDNA. However, the clinical effect of eradicating HBV cccDNA using IFN-α is not as proficient as expected, and it is not well understood yet [67].

A site-specific pegylated recombinant human IFN-β (TRK-560) can significantly suppress the production of extracellular HBsAg and intracellular HBV replication intermediates. The reduction in HBV DNA and intracellular HBV cccDNA levels via IFN-β treatment was found to be significantly higher than that via PEG-IFN-α2a treatment. Moreover, IFN-β exhibited a stronger antiviral potency via higher inductions of IFN-stimulated genes and stronger stimulation of immune cell chemotaxis than that of PEG-IFN-α2a, thus suggesting a potential role of IFN-β in the development of more effective anti-HBV agents [68].

Treatment with IFN-γ can suppress both HBV propagation and transcription. IFN-γ stimulation induced both IFN-γ and IFN-α signaling activation, thereby regulating HBV cccDNA. IFN-γ decreased HBV production via Janus kinase/signal transducer and activator of transcription signaling and IFN-stimulated genes. IFN-γ suppressed HBV propagation and transcription by activating specific intracellular signaling pathways [69]. IFN-γ induced HBV cccDNA deamination and interfered with its stability. HBV-specific T cells inhibited HBV replication and reduced HBV cccDNA through IFN-γ secretion. Consequently, blocking IFN-γ after T cell stimulation can prevent the loss of HBV cccDNA. Deprivation of HBV cccDNA requires nuclear APOBEC3 deaminase activation via the cytokines. Therefore, IFN-γ can reduce HBV cccDNA levels by inducing deamination and subsequent HBV cccDNA decay [70].

#### 2.3.4. ISG20

IFNs can diminish HBV cccDNA via APOBEC3-mediated deamination. Only APOBEC3A overexpression is not sufficient to reduce HBV cccDNA in infected cells, as it requires additional treatment of the hepatocytes with IFNs, thus suggesting the involvement of an interferon-stimulated gene (ISG) in HBV cccDNA degradation. ISG20, as the only type I and II IFN-induced nuclear protein with annotated nuclease activity, localizes to the nucleoli of IFN-stimulated hepatocytes and is enriched on deoxyuridine-containing single-stranded DNA that mimics transcriptionally active APOBEC3A-deaminated HBV DNA. Notwithstanding, ISG20 expression was detected in human livers in acute, self-limiting hepatitis but not in CHB, where its depletion mitigated the IFN-induced loss of HBV cccDNA. More importantly, the co-expression of ISG20 and APOBEC3A is sufficient to diminish HBV cccDNA. Hence, non-cytolytic HBV cccDNA decline requires the concerted action of a nuclease and a deaminase. It suggests that ISGs may be explored for HBV elimination [71].

#### 2.3.5. MX2 (or MxB)

The protein MX2 (MxB), an important IFN-α-inducible effector, reduced HBV RNA levels by downregulating the synthesis of viral RNA and also potently suppressed HBV infection by indirectly impairing HBV cccDNA formation. Namely, it can reduce the amount of HBV cccDNA in HBV-infected cells by indirectly impairing the conversion of HBV rcDNA into HBV cccDNA rather than by destabilizing existing HBV cccDNA. MX2 may represent a novel intrinsic HBV inhibitor that could have therapeutic potential and be useful for improving the understanding of the complex biology of HBV and the anti-HBV mechanisms of IFN-α [72].

#### 2.3.6. TGF-β

Research has shown that transforming growth factor-β (TGF-β) induced HBV cccDNA degradation and hypermutation via activation-induced cytidine deaminase (AID) deamination activity in hepatocytes. The suppression of HBV cccDNA by TGF-β was abrogated when the activity of uracil-DNA glycosylase (UNG) or AID was absent, which indicates that the UNG-mediated excision of uracil and AID deamination act together to degrade HBV cccDNA. Moreover, the HBc protein promoted the interaction between viral cccDNA and AID. Collectivity, it indicates a new mechanism to restrict HBV ccDNA by TGF-β under innate immunity, thereby suggesting a novel approach for eliminating viral cccDNA [73].

#### 2.3.7. HSPA1 Inhibitors

Recent research has indicated that enhanced HBV cccDNA amplification may occur under several selected pathobiological conditions, such as cellular stress, to subvert the dilution or elimination of HBV cccDNA and thereby maintain the persistence of HBV infection. Suppression of heat shock protein family A member 1 (HSPA1)-enhanced HBV cccDNA amplification under the pathobiological conditions could facilitate the complete elimination of HBV cccDNA and cure patients with CHB [74].

#### 2.3.8. CaMKII Activators

Ca^2+^/calmodulin-dependent protein kinase II (CaMKII), which is involved in the calcium signaling pathway, is an important regulator of cancer cell proliferation, motility, growth, and metastasis. HBV replication can suppress CaMKII activity. Conversely, CaMKII upregulation may inhibit HBV replication from HBV cccDNA through AMPK and the AKT/mTOR signaling pathway. Thus, the overexpression or activation of CaMKII may be a new therapeutic target against chronic HBV infection [75].

#### 2.3.9. SART1

In the antiviral process of IFN-*α* against HBV, spliceosome-associated factor 1 (SART1) regulated IFN-mediated antiviral activity via ISG expression and JAK-STAT signaling. The key role of SART1 in the IFN-mediated anti-HBV response provides a new insight into the understanding of variation in response to treatment with IFNs for CHB patients [76]. Importantly, SART1 is a new host factor that suppresses the transcription of HBV cccDNA. In addition to its effect on IFN-stimulated genes, SART1 exerted potent anti-HBV activity by inhibiting the expression of HNF4α, which is essential for HBV cccDNA transcription [77].

### 2.4. Gene-Editing Targeting/Attenuating HBV cccDNA

#### 2.4.1. HBV-Specific CRISPR/Cas9 Systems

Recently, the clustered regularly interspaced short palindromic repeat (CRISPR)/CRISPR-associated protein 9 (Cas9) system was reported to directly target HBV cccDNA and exert antiviral effects. HBV-specific CRISPR-Cas9 systems were showed to be able to effectively mediate the disruption of HBV cccDNA. The designed CRISPR/Cas9 system can accurately and efficiently target HBV cccDNA and suppress HBV replication [78,79]. Although the CRISPR/Cas9 system can specifically target HBV cccDNA for decay, the off-target issue of CRISPR/Cas9 nucleases in the human genome limits its utility in clinical practice. However, the CRISPR/Cas9 system is still a comparatively safe system with high anti-HBV activity, and it can target many HBV variants [80,81]. Additionally, in destroying the HBV genome and inhibiting HBV replication, a synergistic effect was exerted between HBV-specific gRNAs and miR-HBV in gRNA-miR-HBV-gRNA ternary cassettes. More importantly, combining CRISPR/Cas9 with the RNA interference (RNAi) approach, the HBV-specific gRNAs indicated a potent effect on HBV cccDNA destruction [82]. This suggests that Cas9-mediated base editing may be an effective means to cure CHB via permanent inactivation of integrated HBV DNA and cccDNA without double-strand breaks of the host genome [83]. Currently, EBT-107 from Excision Biotherapeutics is at the preclinical stage.

#### 2.4.2. Sequence-Specific ARCUS Nuclease

Targeting the HBV genome using a specifically engineered ARCUS nuclease (ARCUS-POL) is a potential therapeutic approach. Transient expression of ARCUS-POL produced substantial reductions in HBV cccDNA and HBsAg in HBV-infected hepatocytes. A significant reduction in high on-target indel frequency and total adeno-associated virus (AAV) copy number were observed in both a non-human primate model containing a portion of the HBV genome and an episomal AAV mouse model via systemic administration of lipid nanoparticles containing ARCUS-POL mRNA. Circulating surface antigen was durably decreased by 96% in the mouse supporting HBsAg expression. Taken together, these findings support the gene-editing approach for HBV cccDNA elimination [84]. Currently, the ARCUS platform-based PBGENE-HBV from Precision BioSciences is at the preclinical stage. Additionally, replication-incompetent AAV-based vectors are non-pathogenic viral particles used to deliver therapeutic genes to treat multiple disorders. Furthermore, the safety of AAV-based vector administration has been demonstrated in non-human primates [85].

#### 2.4.3. siRNAs for Silencing Viral RNA

RNA interference agents have the potential to impact the entire viral life cycle by reducing all virus-produced mRNA [86]. Research has revealed that siRNA could serve as an efficient alternative anti-HBV agent, as it exhibited better inhibitive effects on antigen expression and viral replication. More importantly, the siRNA markedly suppressed HBV cccDNA amplification [87]. Even though RNAi has been proven to be able to silence the target gene expression and thereby decrease HBV replication, siRNA is susceptible to be degraded by RNA enzymes, making it difficult to successfully deliver and lacking tissue-specific targeting. Additionally, PreS/2-21-directed nanoparticles loaded with anti-HBV gene therapy drugs are stable, safe, and highly targetable, with potent inhibitive effects on HBV DNA, pgRNA, and HBV cccDNA. Hence, it is a promising approach for the treatment of CHB patients [88]. ARC-520 is a first-generation RNA interference drug, but its clinical study was stopped due to its hepatotoxicity [89,90]. Other siRNA drugs (VIR-2218, AB-729, RG6346, etc.) have advanced into phase II clinical trials for CHB treatment.

## 3. Discussion

HBV is a major human pathogen that remains a worldwide public health concern. Its infection is a leading cause of liver diseases with a high burden worldwide. Therefore, potentially curative agents are of high importance. Following viral infection, the existence of extraordinary stable HBV cccDNA in the nuclei of HBV-infected cells serves as a template for HBV replication and forms a reservoir for persistent HBV infection. Therefore, HBV cccDNA is an obstacle to achieving complete clearance of HBV during antiviral treatment of CHB patients. The current strategy against HBV relies on interferon and nucleos(t)ide-type drugs, with the limitation of functional cure. Although IFN-α can target HBV cccDNA theoretically, its clinical effect is unsatisfactory on HBV cccDNA [67]. All approved nucleos(t)ide reverse transcriptase inhibitors are effective in suppressing HBV DNA synthesis via their inhibition of HBV polymerase. However, nucleos(t)ide analogues have no direct effect on viral cccDNA, despite there being a significant decline in HBV DNA [91]. Generally, current anti-HBV therapies have little effect on viral cccDNA and fail to eliminate HBV. Curing CHB requires a novel strategy for purging HBV cccDNA from patients. Therefore, there is an urgent need to develop novel therapeutic agents targeting or attenuating HBV cccDNA formation and maintenance. Since a true cure of CHB requires the elimination of the cccDNA from infected cells, the comprehension of the state of the art and the challenges in efforts to eliminate or inactivate HBV cccDNA by different mechanisms, approaches, and strategies appears necessary to achieve HBV eradication.

The ultimate goal of CHB treatment is to eliminate viral cccDNA from the hepatocyte nucleus for eradicating infections that are initiated or reinitiated by HBV cccDNA [92]. Viral cccDNA is the main element of the HBV replication cycle, as it serves as the transcription template of all viral RNAs (including pgRNA) and subsequently forms the progeny HBV DNA genomes. HBV cccDNA is either amplified from encapsidated rcDNA or originated from new incoming virions from the hepatocyte cytoplasm. The reduction in intranuclear HBV cccDNA often occurs after the destruction of HBV-infected hepatocytes, apoptosis of hepatocytes, non-cytolytic immune responses, and compensatory cell proliferation [93]. Although elimination of intranuclear HBV cccDNA is difficult to achieve with the current available drugs, a cure is possible, in theory, via the immune system and hepatocyte turnover. Nevertheless, the half-life of HBV cccDNA is relatively long because hepatocytes have a long half-life (more than six months or even years). Therefore, the elimination of viral cccDNA via hepatocyte turnover is not a major means of clearance [94]. Research advances in the HBV replication cycle facilitate to develop novel antiviral agents by targeting multiple steps of viral replication with different mechanisms. For instance, HBx binding to cccDNA plays a key role in the activation of HBV gene transcription, leading to efficient virus replication. Conversely, the suppression of HBx binding to cccDNA leads to inhibition of viral gene transcription and virus replication by modifying the epigenetic regulation of viral cccDNA function with host restriction factors. HBc inhibitors targeting cccDNA-bound HBc may alter the epigenetics of HBV cccDNA, and in combination with HBx inhibitors, this could theoretically lead to a stronger synergistic effect on viral cccDNA transcriptional silencing and HBV replication [8]. Therefore, although HBV cccDNA is inherently stable, it is silenced in function via epigenetic modifications with epigenetic drugs, which may represent a new class of antivirals for CHB treatment.

Past studies have demonstrated that it is possible to degrade HBV cccDNA in the nuclei of hepatocytes with minimal hepatotoxicity effects. The programmable RNA-guided DNA endonucleases (including the CRISPR/Cas9 system) have exhibited their potential to serve as an effective tool to deplete the cccDNA pool in HBV-infected hepatocytes. Moreover, the upregulation of APOBEC3A/B deaminase by several cytokines (including IFN-α, IFN-γ, tumor necrosis factor-α, and lymphotoxin-β receptor agonists) can cause the partial degradation of HBV cccDNA without hepatotoxicity [64]. These targets aim to prevent HBV cccDNA formation through destroying or damaging it. The CRISPR/Cas9-based approach is one of the most promising strategies to achieve a complete sterilizing cure of CHB, as it can efficiently destroy HBV-expressing templates from genotypes A to D without apparent cytotoxicity [95]. Moreover, developing new anti-HBV agents based on CRISPR/Cas9 ribonucleoprotein complexes could substantially decrease the duration of CHB therapy and have the potential to achieve complete elimination of HBV infection [96]. However, the key obstacle against the CRISPR/Cas9 system in clinical utilization is regarding the off-target problem of gene-editing applications and their delivery efficiency in vivo. In addition, cutting integrated HBV genomes via CRISPR/Cas9 also raised serious concern due to their potential risk of genome instability [97]. Further research needs to be carried to eliminate the possibility of expressing an off-target effect that causes damage to host DNA. As an alternative way, CRISPR/Cas9 nickase only causes single-strand DNA breaks rather than dsDNA breaks. Thus, a pair of properly spaced guide RNAs can only cause dsDNA breaks, and the risk of permanent DNA damage due to the off-target effect could be greatly reduced [98]. Additionally, engineered site-specific nucleases and RNAi therapeutics have the potential to eliminate HBV cccDNA or silence its transcription.

Induction of APOBEC3B expression can lead to HBV cccDNA decay and its induction is transcriptionally regulated via NF-κB signaling and post-transcriptionally downregulated by hsa-miR-138-5p expression. Timely controlled APOBEC3B-mediated HBV cccDNA decay occurs independently of the transcriptional activity of HBV cccDNA. Consequently, APOBEC3B-mediated HBV cccDNA decay may provide an efficient alternative to target HBV infection [99]. APOBEC3G displays broad antiretroviral activity and can also suppress HBV replication. APOBEC3G and APOBEC3F can downregulate the production of replication-competent hepadnaviral nucleocapsids [100]. Based on the inhibition of APOBEC/AID on HBV replication by deaminating and destroying cccDNA, a CRISPR-activation-based approach (CRISPRa) can induce transient APOBEC/AID overexpression for eliminating HBV cccDNA. Coupling CRISPRa RNPs with att-sgRNA technology for simultaneous, tunable, and transient regulation of complex antiviral programs paves the way for using CRISPRa approaches as a novel curing strategy for suppressing HBV replication [101]. 

Collectively, a standardized cure for CHB requires the approaches that degrade or silence HBV cccDNA. (1) Indirect HBV cccDNA epigenetic silencing: small molecules targeting host histone deacetylases, demethylases, and acetyltransferases can interrupt the normal epigenetic regulation of HBV gene expression and thereby silence HBV cccDNA transcription. For instance, the oral (H3K4me3:H3) KDM5 demethylase inhibitor GS-5801 can silence the transcription of HBV cccDNA, but its clinical development was stopped due to safety concerns [13,102]. EYP001, as a farnesoid X receptor agonist, is a potent inhibitor of HBV cccDNA transcription in clinical development [52,53]. Other candidate drugs that block HBV cccDNA transcription in the discovery stage, such as AG inhibitors, which suppress the nuclear transcript Sp1, and dicoumarol, a competitive nicotinamide adenine dinucleotide phosphate quinone oxidoreductase, act as an inhibitor of HBx expression [11,23]. The HBx protein modifies the epigenetic regulation of HBV cccDNA function. Small molecules, siRNAs, and gene-editing approaches to knockdown HBx expression are all being evaluated in preclinical studies. (2) Direct HBV cccDNA silencing: combining Cas9 with guide RNAs targeting the conserved HBV sequences led to a dramatical reduction in HBV cccDNA and all HBV proteins [103]. The major challenges of translating gene editing in clinical trials are the need to achieve 100% efficiency of delivery to the HBV-infected hepatocytes and to mitigate the long-term risks of possible chromosomal translocation following the editing of integrated HBV sequences. Peg-IFNα is involved in both the transcription and degradation of HBV cccDNA [64,104]. Peg-INFα induced the degradation of HBV cccDNA via upregulation of APOBEC3A and 3B cytidine deaminases. It also blocked HBV cccDNA transcription by inducing viral cccDNA-bound histone hypoacetylation. Notably, the formation and maintenance of HBV cccDNA pools may be partially suppressed by baseline expression of host inhibitive factors, e.g., APOBEC3A and 3B [105]. 

The primary role of the HBx protein is to promote the transcription of the HBV genome. HBx fulfills this task by only enhancing transcription from extrachromosomal DNA templates. However, the structural maintenance of the chromosome 5/6 (SMC5/6) complex as a restriction factor selectively blocks extrachromosomal DNA transcription. Therefore, HBx relieves the inhibition to allow productive HBV gene expression by destroying this complex [106]. Interaction of HBx with the DDB1-CUL4-ROC1 (CRL4) E3 ligase is critical for the function that HBx activates gene expression from the HBV cccDNA genome. Thus, this indicates that HBx’s primary function is to degrade SMC5/6, which restricts HBV replication by repressing HBV gene expression [107]. HBV cccDNA is silenced naturally during infection by binding of the SMC5/6 complex to HBV cccDNA, and HBV antagonizes this silencing with the HBx protein that binds to SMC5/6 and triggers its proteasomal degradation [108]. Additionally, to study the impact of peg-IFNα on the HBx protein and SMC5/6, HBV-infected human liver chimeric mice were treated with peg-IFNα for six weeks, and then peg-IFNα was withdrawn and the mice received the entry inhibitor bulevirtid for six weeks following treatment. The results showed that blocking viral entry with bulevirtid hindered intrahepatic viral rebound, reduced cccDNA loads, and enhanced IFN-stimulated genes. Furthermore, peg-IFNα abrogated HBx production in a substantial amount of hepatocytes, leading to the prompt reappearance of SMC5/6 and cccDNA silencing. This suggests that therapeutic strategies targeting HBx and aiming at restoring SMC5/6 function in HBV-infected hepatocytes may reduce the progression of hepatitis B, and that the combinations of IFN with bulevirtid could contribute to cure CHB infections [109].

Targeting host factors to achieve functional silencing of HBV cccDNA may represent a new strategy for the treatment of CHB infection. To assess the effects of Jumonji C domain-containing (JMJD2) protein subfamily JMJD2A–2D proteins on HBV replication, lentivirus-based RNAi was used to suppress the isoforms JMJD2A–2D expression in HBV-infected cells. The results showed that HBV cccDNA transcription and HBV replication were inhibited via downregulation of JMJD2D or 5C-8-HQ-mediated JMJD2D inhibition. Mechanistically, JMJD2D sustained HBx stability by inhibiting the TRIM14-mediated ubiquitin–proteasome degradation pathway and acted as a key co-activator of HBx to augment HBV replication. Thus, this indicates that JMJD2D may be used as a potential diagnostic biomarker and promising drug target against CHB infection [110].

Viral cccDNA, as the transcriptional template of HBV, interacts with both host and viral proteins to form minichromosomes in the nuclei of infected cells and is resistant to antiviral agents. The host factors involved in HBV cccDNA transcriptional regulation is expected to be able to provide a new approach for CHB therapy. Protein arginine methyltransferase 5 (PRMT5), as an effective restrictor of HBV transcription and replication, restricts HBV replication via a two-part mechanism, including epigenetic suppression of HBV cccDNA transcription and interference with pgRNA encapsidation [111]. These findings improved the understanding of epigenetic regulation of HBV transcription and host–HBV interactions, thus providing new insights into targeted therapeutic intervention [112]. In addition, long non-coding RNAs (lncRNAs) were found to be involved in mediating the interaction of host factors with various viruses. For instance, LINC01431 was proved to be a novel host restriction factor for HBV transcription. Mechanically, LINC01431 blocked the HBx-mediated type I protein arginine methyltransferase (PRMT1) ubiquitination and degradation processes by competitively binding with PRMT1 [113]. Consequently, LINC01431 increased the occupancy of PRMT1 on HBV cccDNA, leading to enhanced H4R3me2a modifications and decreased acetylation of HBV cccDNA-bound histones, thereby suppressing HBV cccDNA transcription. In turn, to facilitate viral replication, HBV transcriptionally repressed LINC01431 expression via HBx-mediated inhibition of transcription factor Zinc fingers and homeoboxes 2. Taken together, this indicates that LINC01431 is a novel epigenetic regulator of HBV cccDNA minichromosomes and highlights a feedback loop of HBx-LINC01431-PRMT1 in HBV replication, which provides a promising therapeutic target for HBV infection [113]. 

Recently, it was shown that the degradation of HBV cccDNA alone seems to be insufficient to eliminate HBV infection, as it rebounds and results in de novo formation of cccDNA via rcDNA. CRISPR-Cas9-mediated inactivation of HBV cccDNA was demonstrated to be insufficient for curing HBV infection. HBV replication rapidly rebounds after CRISPR-Cas9 ribonucleoprotein (RNP) degradation, and the HBV cccDNA pool is reestablished due to de novo formation of HBV cccDNA from rcDNA. However, depleting HBV rcDNA in infected cells with lamivudine prevents cccDNA re-establishment and promotes the resolution of HBV infection via a single dose of CRISPR-Cas9 RNPs [114]. This suggests that blocking HBV cccDNA replenishment and re-establishment from HBV rcDNA conversion is critical for completely eliminating HBV from the infected cells. Therefore, the combination of the degradant of HBV cccDNA with the inhibitor of rcDNA formation or rcDNA to cccDNA conversion may be even more useful.

Generally, there is, so far, no therapeutic that can completely cure already infected HBV patients due to the inability of humans to eliminate viral cccDNA and the inadequate host immune response to HBV infection [115]. Considering that the inactivation/inhibition or elimination of HBV cccDNA is the core goal of anti-HBV therapeutic strategies, small molecules, polypeptides/proteins, and siRNA, as well as gene-editing approaches by targeting or attenuating HBV cccDNA, are the main means to achieve this goal (Table 1). Nevertheless, all of the above means are being evaluated in preclinical studies or clinical trials. Currently, there are still some challenges: (1) The formation and regulation of HBV cccDNA, especially the conversion of HBV rcDNA into cccDNA, are not fully clarified yet. (2) The absence of robust models for the study of HBV cccDNA due to the fact that it is difficult for HBV to infect liver cells in vitro and in vivo. (3) Direct targeting of HBV cccDNA is still a challenge, especially with no good means of blocking HBV cccDNA replication [111]. 

In conclusion, eliminating or silencing HBV cccDNA in infected cells and breaking host immune tolerance to HBV infection are two ways to eradicate HBV in CHB patients [116]. However, no breakthrough has been made yet in small molecules, polypeptides, and proteins targeting/inactivating HBV cccDNA, although some candidates have advanced into the phase of clinical trials. What is more important, no outstanding candidate directly targets HBV cccDNA. The overwhelming majority of the candidates indirectly target HBV cccDNA by inactivating/inhibiting its function. Relatively speaking, as two first-in-class agents, CCC_R08 and nitazoxanide may be some of the most promising agents to clear HBV infection in small molecule compounds [14,85]. Additionally, CRISPR-Cas9 systems can directly target HBV cccDNA for decay and demonstrate significant anti-HBV activity. Consequently, gene-editing approaches targeting HBV cccDNA may be one of the most promising means to achieve the core goal of anti-HBV therapeutic strategies. Overall, more studies on HBV infection need to be carried out to overcome these challenges.

## Figures and Tables

**Table 1:** Classification and mechanisms of the main agents/approaches for targeting or attenuating HBV cccDNA.

Classification	Agent/Approach against HBV	Mechanism	R&D phase	References
Small molecules for epigenetically silencing HBV cccDNA	Rapamycin	Targeting HBx to block HBV cccDNA transcription.	Preclinical	[9]
	Dicoumarol	Blocking cccDNA transcription by promoting HBx degradation.	Preclinical	[11]
	GS-080 and GS-5801	Silencing cccDNA transcription by histone lysine demethylase inhibitor.	Phase 1b (GS-5801)	[12,13]
Small molecules for suppressing HBV cccDNA	CCC_R08	Specifically reduce cccDNA levels.	Preclinical	[14]
	CCC-0975 and CCC-0346	Interfering primarily with rcDNA conversion into cccDNA.	Preclinical	[15]
	Junceellolide C and junceellolide B	Transcription inhibitors of cccDNA by inhibiting HBV RNA transcription.	Preclinical	[16,17]
	Compound **59**	HBV cccDNA and antigen reducers.	Preclinical	[18]
	Epigallocatechin gallate (EGCG)	Inhibiting HBV entry and HBV cccDNA production by impairing HBV replicative intermediates of DNA synthesis.	Preclinical	[19,20,21]
	Ivermectin	Suppressing the production of cccDNA via depletion of KPNA1-6 and the nuclear import of HBV by inhibiting KPNA2.	Preclinical	[22]
	1-[3-(4-tert-butylcyclohexyl)oxy-2-hydroxypropyl]-2,2,6,6-tetramethylpiperidin-4-ol	Reducing HBV cccDNA production via interaction with the nuclear transcription factor Sp1.	Preclinical	[23]
	NJK14047	Decreasing pgRNA and HBV cccDNA by inhibiting the selective host factor P38 mitogen-activated protein kinase (MAPK).	Preclinical	[24]
	3,4-di-*O*-CQA and 3,5-di-*O*-CQA	Blocking the replenishment of HBV cccDNA by reducing the stabilization of HBV core protein via HO-1 overexpression.	Preclinical	[25,26]
	ABI-H2158	Inhibiting HBV replication by blocking pgRNA encapsidation, and it also potently blocked the formation of cccDNA.	Terminated in Phase II due to its hepatotoxicity	[27]
	Pimobendan (Pim)	As a transcription inhibitor of cccDNA through suppressing HBV promoters to reduce HBV RNA levels and HBsAg production.	Preclinical	[28]
	ABI-H0731	(1) Preventing HBV pgRNA encapsidation and subsequent DNA replication by targeting the HBV core protein; and (2) preventing new cccDNA formation by disrupting incoming nucleocapsids, causing the premature release of rcDNA before delivery to the nucleus.	Phase II	[29]
	UCN-01	Decreasing cccDNA levels as an inhibitor with a broad spectrum activity for phosphorylated protein kinase C (PKC) and cyclin-dependent kinase (CDK) proteins.	Preclinical	[30]
	AZD-5438	Blocking intracellular cccDNA synthesis as a potent inhibitor of CDK.	Preclinical	[30,31]
	Tazarotene	Repressing HBV cccDNA transcription and its inhibition on HBV, in part, through RARβ.	Preclinical	[32]
	Peretinoin	(1) Enhancing the binding of histone deacetylase 1 (HDAC1) to cccDNA in the nucleus and negatively regulating HBV transcription. (2) Activating HDAC1 and thereby suppressing HBV replication by inhibiting the sphingosine metabolic pathway.	Preclinical	[33]
	Curcumin	Reducing intracellular HBV DNA replication intermediates and HBV cccDNA via downregulation of cccDNA-bound histone acetylation.	Preclinical	[34]
	Punicalagin, punicalin, and geraniin	Inhibiting HBV cccDNA production via a dual mechanism through preventing the formation of cccDNA and promoting cccDNA decay.	Preclinical	[35]
	Osalmid and YZ51	Inhibiting HBV DNA and cccDNA synthesis by targeting the ribonucleotide reductase (RR) small subunit M2 (RRM2).	Preclinical	[36]
	Irbesartan	As a NTCP-interfering molecule, inhibiting HBV cccDNA formation post-uptake prior to the cccDNA formation step.	Preclinical	[37]
	MLN4924	As a potent and selective NEDD8-activating enzyme inhibitor, inhibiting cccDNA transcription by blocking cullinneddyltion and activating ERK to suppress the expression of several transcription factors required for HBV replication, including HNF1α, C/EBPα, and HNF4α.	Preclinical	[38,39]
	Rnase H inhibitors 110, 1133, and 1073	As Rnase H inhibitors, suppressing cccDNA formation by blocking amplification of HBV cccDNA, which suppresses events downstream of cccDNA formation.	Preclinical	[41]
	Clevudine/ATI-2173	As a first-generation ASPIN and a novel next-generation ASPIN, both have demonstrated the potential ability to reduce cccDNA biomarkers.	Approved 2006 in S. Korea/ Phase Ib	[42]
	HAP_R01	As a structurally distinct heteroaryldihydropyrimidine (HAP)-type CpAM, inhibiting cccDNA formation by perturbing capsid integrity of incoming virus particles and reducing their infectivity, as well as preventing HBV capsid assembly.	Preclinical	[43,44,45,46]
	CDK9 inhibitor FIT-039	As a CDK9 inhibitor, its antiviral activity at an early phase of viral infection and reducing cccDNA in HBV-infected cells.	Preclinical	[50]
	Olaparib	As a PARP inhibitor, increasing the reductions in pgRNA and cccDNA levels induced by HBV-CRISPR. The suppression of the NHEJ-mediated DNA repair machinery enhances the effect of CRISPR targeting cccDNA.	Preclinical	[51]
	Vonafexor (EYP001)	As a FXR agonist, it is a potent inhibitor of cccDNA transcription.	Phase Ib	[52,53]
	Nitazoxanide and tizoxanide	Silencing the transcription of HBV cccDNA and decreasing viral cccDNA levels slightly by targeting HBx–DDB1 interactions and significantly restoring Smc5 protein levels.	Phase I	[55,56,57,58]
Polypeptides/proteins for inhibiting HBV cccDNA	Bulevirtide (myrcludex B/hepcludex)	As the first entry inhibitor that can inactivate HBV and HDV receptors, it can inhibit the amplification of cccDNA as well as the spread of intrahepatic infection.	Approved in 2023 in the EU; Phase III in the USA	[59,60,61,62]
	Pam3SCK4	As a TLR-2 agonist and potential immune stimulator, decreasing HBV RNA production (inhibition of synthesis and acceleration of decay) and cccDNA levels through the TLR1/2- NF-κB canonical-pathway.	Preclinical	[63]
	IFN-α2b/PEG-IFN-α2a	(1) HBV cccDNA molecule is degraded non-cytolytically by IFN-α that upregulates APOBEC3A and 3B. (2) IFN-α epigenetically regulates the HBV cccDNA minichromosome by modulating the GCN5-mediated succinylation of histone H3K79 to clear HBV cccDNA. (3) TNF-α-induced deamination of cccDNA and interference with its stability.	Approved 1991; approved 2005 in the USA	[64,65,66,67,70]
	IFN-β (TRK-560)	IFN-β treatment shows a stronger potency in intracellular HBV cccDNA reduction via higher levels of induction of interferon-stimulated genes and stronger stimulation of immune cell chemotaxis than PEG-IFN-α2a.	Preclinical	[68]
	IFN-γ	(1) IFN-γ decreases HBV production through Janus kinase/signal transducer and activator of transcription signaling and interferon-stimulated genes. (2) IFN-γ reduces HBV cccDNA levels in hepatocytes by inducing deamination and subsequent cccDNA decay.	Preclinical	[69,70]
	ISG20	As the only type I and II interferon-induced nuclear protein with annotated nuclease activity, ISG20 depletion mitigates the interferon-induced loss of cccDNA, and co-expression with APOBEC3A is sufficient to diminish cccDNA.	Preclinical	[71]
	MX2 (or M xB)	As an IFN-α-inducible effector, reducing the amount of HBV cccDNA by indirectly impairing the conversion of rcDNA into cccDNA rather than by destabilizing existing cccDNA.	Preclinical	[72]
	TGF-β	Inducing viral cccDNA degradation and hypermutation via AID deamination activity in hepatocytes.	Preclinical	[73]
	HSPA1 inhibitors	Inhibiting HSPA1-enhanced cccDNA amplification under selected pathobiological conditions to facilitate the elimination of cccDNA and cure CHB.	Preclinical	[74]
	CaMKII activator	Suppressing HBV replication from cccDNA via the activation or overexpression of CaMKII or upregulation of CaMKII activity.	Preclinical	[75]
	SART1	(1) Regulating IFN-mediated antiviral activity through JAK-STAT signaling and ISG expression in the antiviral activity of IFN-α against HBV. (2) As a host factor suppressing HBV cccDNA transcription, it exerts anti-HBV activity by suppressing HNF4α expression, which is essential for the transcription of HBV cccDNA.	Preclinical	[76,77]
Gene-editing approaches targeting/attenuating HBV cccDNA	HBV-specific CRISPR/Cas9 systems (EBT107)	As a genome-editing tool, it can specifically and accurately target HBV cccDNA to effectively mediate cccDNA disruption.	Preclinical	[78,79,80,81]
	Sequence-specific ARCUS nuclease (PBGENE-HBV)	As a highly specific engineered ARCUS nuclease (ARCUS-POL) targeting the HBV genome, transient ARCUS-POL expression can produce substantial reductions in both cccDNA and HbsAg.	Preclinical	[84]
	siRNAs by silencing viral RNA (VIR-2218, AB-729, and RG6346)	As an alternative anti-HBV agent, it can inhibit HBV cccDNA amplification by silencing the expression of target genes.	Phase II	[87,88,89,90]

## Data Availability

Data are contained within the article.
